# Percutaneous Transvenous Retrograde Embolization of Adhesion-Related Small Bowel Varices: A Case Report

**DOI:** 10.7759/cureus.94009

**Published:** 2025-10-07

**Authors:** Ghanem Mohamed, Sondes Bizid, Hatem Ben Abdallah, Mohamed Riadh Bouali

**Affiliations:** 1 Department of Gastroenterology and Hepatology, Military Hospital of Tunis, Faculty of Medicine of Tunis, University of Tunis El Manar, Tunis, TUN

**Keywords:** bowel adhesion, ectopic varices, ethiodized oil, jejunal varices, lower gastrointestinal bleeding, mesenteric-iliac shunt, n-butyl-2-cyanoacrylate, percutaneous access, portal hypertension, transvenous embolization

## Abstract

Small bowel varices are a distinct subtype of ectopic varices that typically develop following prior abdominal surgery. Diagnostic delays are common because of their deep location within the gastrointestinal tract and the limited awareness among clinicians. Transvenous obliteration is the preferred treatment; however, obtaining a suitable and minimally invasive venous access route remains challenging. We report the case of a 54-year-old woman with advanced-stage primary biliary cholangitis (PBC) and celiac disease who presented with persistent melena requiring multiple transfusions. Endoscopic evaluations failed to identify the bleeding source. Contrast-enhanced computed tomography revealed adhesion-related jejunal varices arising from a mesenteric-iliac shunt. Percutaneous venous access was achieved via the efferent vein coursing within the anterior abdominal wall. Retrograde embolization of the varices was then performed using N-butyl-2-cyanoacrylate mixed with ethiodized oil. The procedure achieved immediate hemostasis, with no recurrence observed during nine years of follow-up. This case underscores three key points: (1) ectopic varices should be considered in any patient with portal hypertension presenting with unexplained gastrointestinal bleeding, particularly after abdominal surgery; (2) cross-sectional imaging is essential for diagnosis, delineation of shunt anatomy, and selection of the optimal treatment and venous access route; and (3) when anatomically feasible, superficial venous puncture should be the preferred approach for transvenous obliteration due to its minimally invasive nature.

## Introduction

Acute variceal bleeding is a severe and life-threatening complication of portal hypertension. Despite advances in pharmacological therapy, endoscopic management, and endovascular interventions such as transjugular intrahepatic portosystemic shunt (TIPS) and transvenous obliteration, six-week mortality remains high at 10%-15% [1,2]. Prognosis depends on the severity of portal hypertension, the stage of liver disease, and the site of the varices [1,2]. While gastroesophageal varices are the most common source of bleeding, varices can also develop outside this region and are classified as ectopic varices [1,2].

Ectopic varices may occur in any part of the gastrointestinal tract, as well as in the gallbladder, bladder, uterus, or vagina [3,4]. They account for approximately 5% of all hemorrhagic events related to portal hypertension, with reported mortality rates as high as 40% [3]. Small bowel varices represent a distinct subtype of ectopic varices, typically arising after abdominal surgery in patients with portal hypertension [3-6]. Diagnostic delays are common due to their deep intraluminal location and limited clinician awareness [3,4]. The management of ectopic varices remains non-standardized, as current evidence is largely limited to case reports and small series [7-10]. Transvenous obliteration is considered the preferred treatment for small bowel varices; however, obtaining suitable and minimally invasive venous access can be challenging [4]. Here, we report a case of adhesion-related small bowel varices successfully treated with transvenous obliteration through an uncommon percutaneous approach.

## Case presentation

A 54-year-old woman with advanced-stage primary biliary cholangitis (PBC) and celiac disease was referred for evaluation of persistent melena. Her past surgical history included umbilical hernia repair. She had been hospitalized for two weeks at a local facility, during which she received multiple blood transfusions. The source of bleeding remained undetermined despite upper and lower gastrointestinal endoscopy.

On examination, her blood pressure was 90/58 mmHg and heart rate 90 beats per minute. There was no ascites or evidence of neurological impairment. Laboratory investigations showed severe anemia with a hemoglobin level of 4.0 g/dL and mild cholestasis. Complete laboratory findings are summarized in Table 1.

**Table 1:** Initial laboratory parameters and reference ranges AST: aspartate aminotransferase, ALT: alanine aminotransferase, ALP: alkaline phosphatase, GGT: gamma-glutamyl transferase, CRP: C-reactive protein, INR: international normalized ratio.

Parameter	Result	Reference Range
Hemoglobin	4.0 g/dL	13-18 g/dL
Platelet count	132 × 10³/μL	150-450 × 10³/μL
Total bilirubin	2.51 mg/dL	<1 mg/dL
AST	109 IU/L	10-60 IU/L
ALT	94 IU/L	10-42 IU/L
ALP	561 IU/L	42-121 IU/L
GGT	662 IU/L	7-64 IU/L
Creatinine	0.54 mg/dL	0.6-1.2 mg/dL
CRP	5.0 mg/L	<8 mg/L
INR	1.12	0.8-1.2
Albumin	32 g/L	35-50 g/L

Repeat esophagogastroduodenoscopy revealed small esophageal varices without signs of recent bleeding. No gastric varices were observed. Colonoscopy demonstrated fresh blood in the lumen but no identifiable bleeding source.

**Figure 1:**
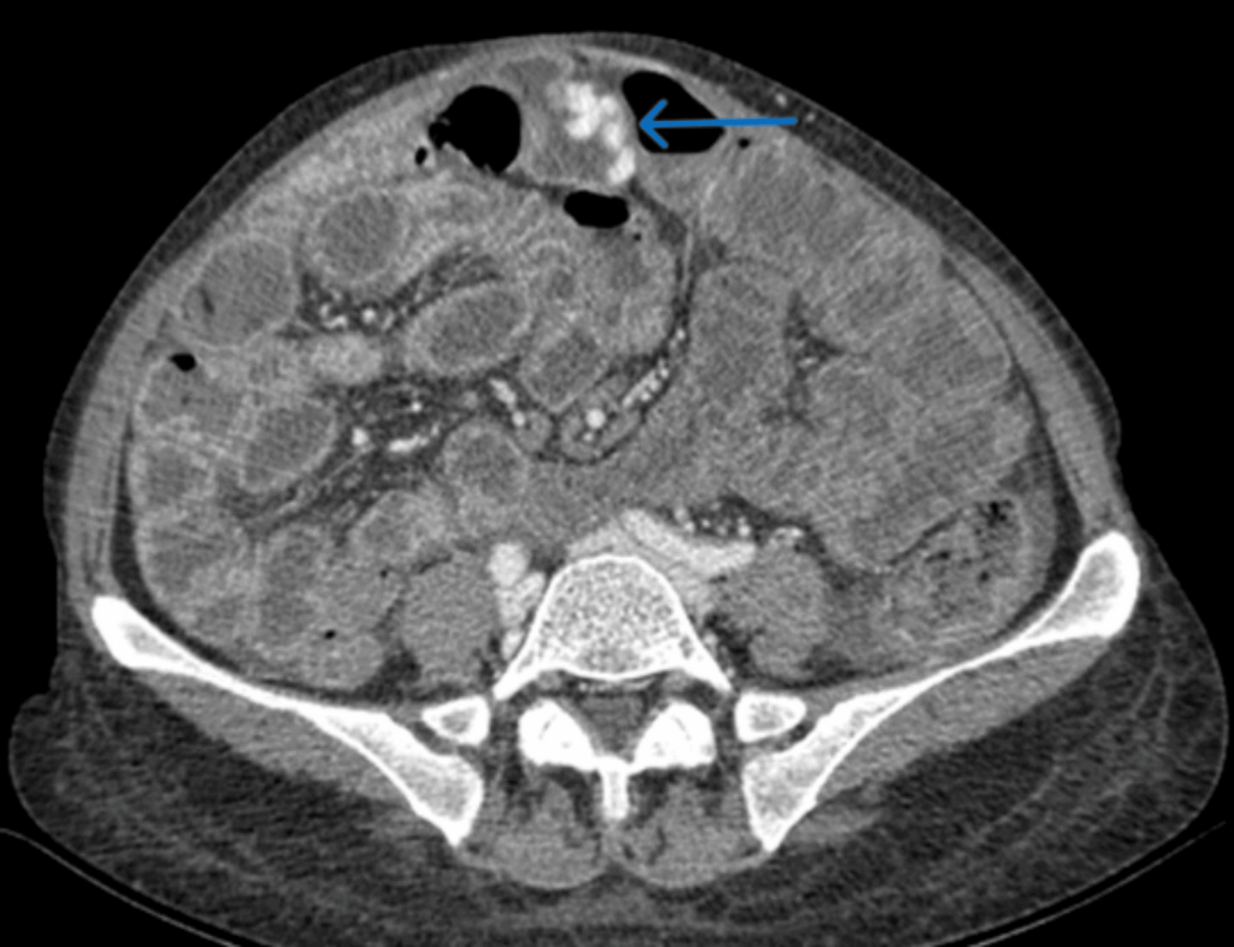
Portal-phase abdominal CT showing jejunal varices (blue arrow)

**Figure 2:**
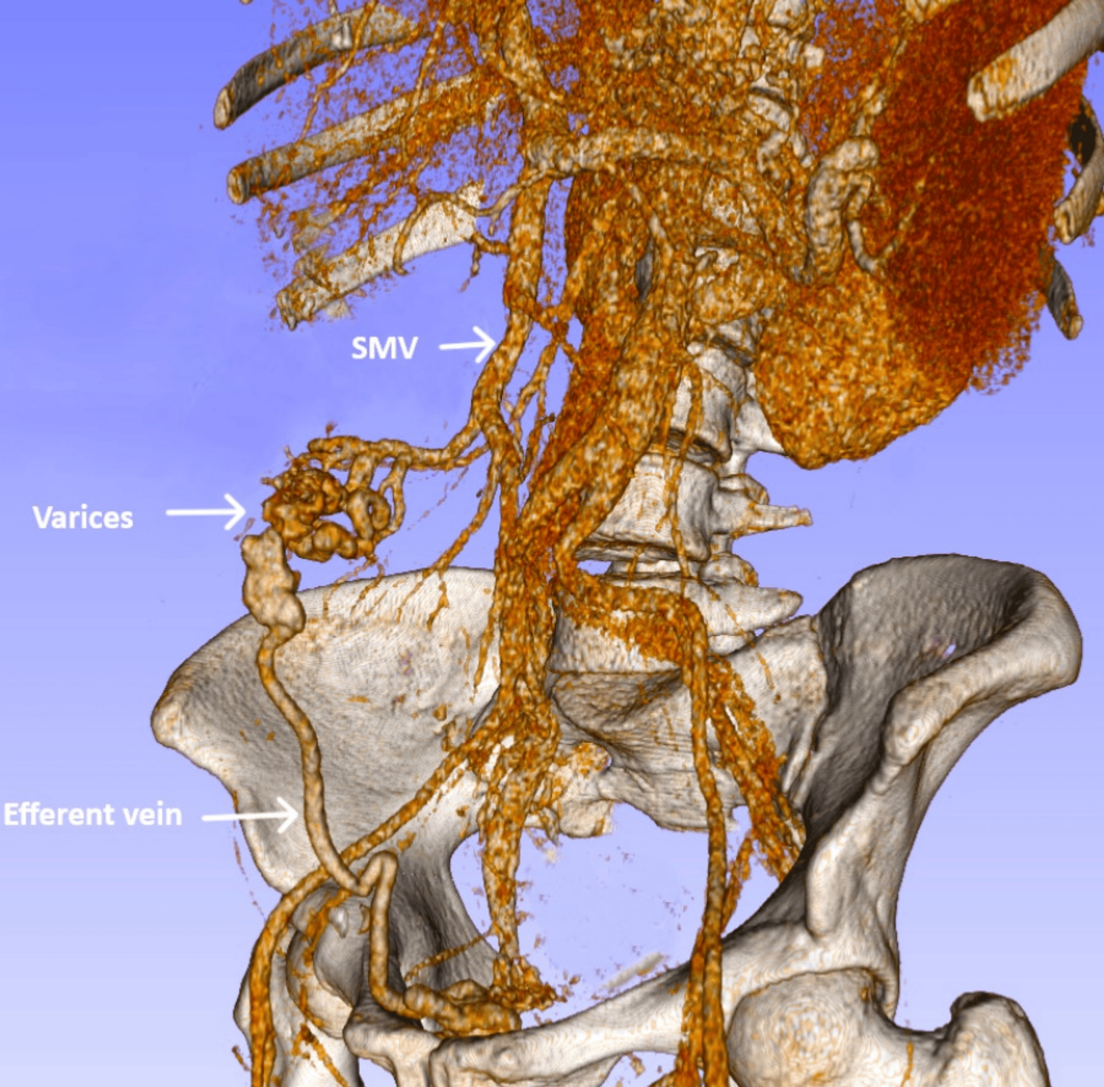
3D reconstruction of the mesenteric-iliac shunt SMV: superior mesenteric vein. Note: This image was created using 3D Slicer v5.8.1 (https://www.slicer.org/).

Contrast-enhanced abdominal CT showed a dilated, tortuous vein within the wall of a jejunal loop adjacent to the anterior abdominal wall (Figure 1). A tributary of the superior mesenteric vein served as the feeding vein, while an anterior abdominal wall vein draining into the internal iliac veins acted as the efferent vein. No contrast extravasation was detected. These findings were consistent with bleeding from adhesion-related jejunal varices forming part of a mesenteric-iliac shunt (Figure 2).

**Figure 3:**
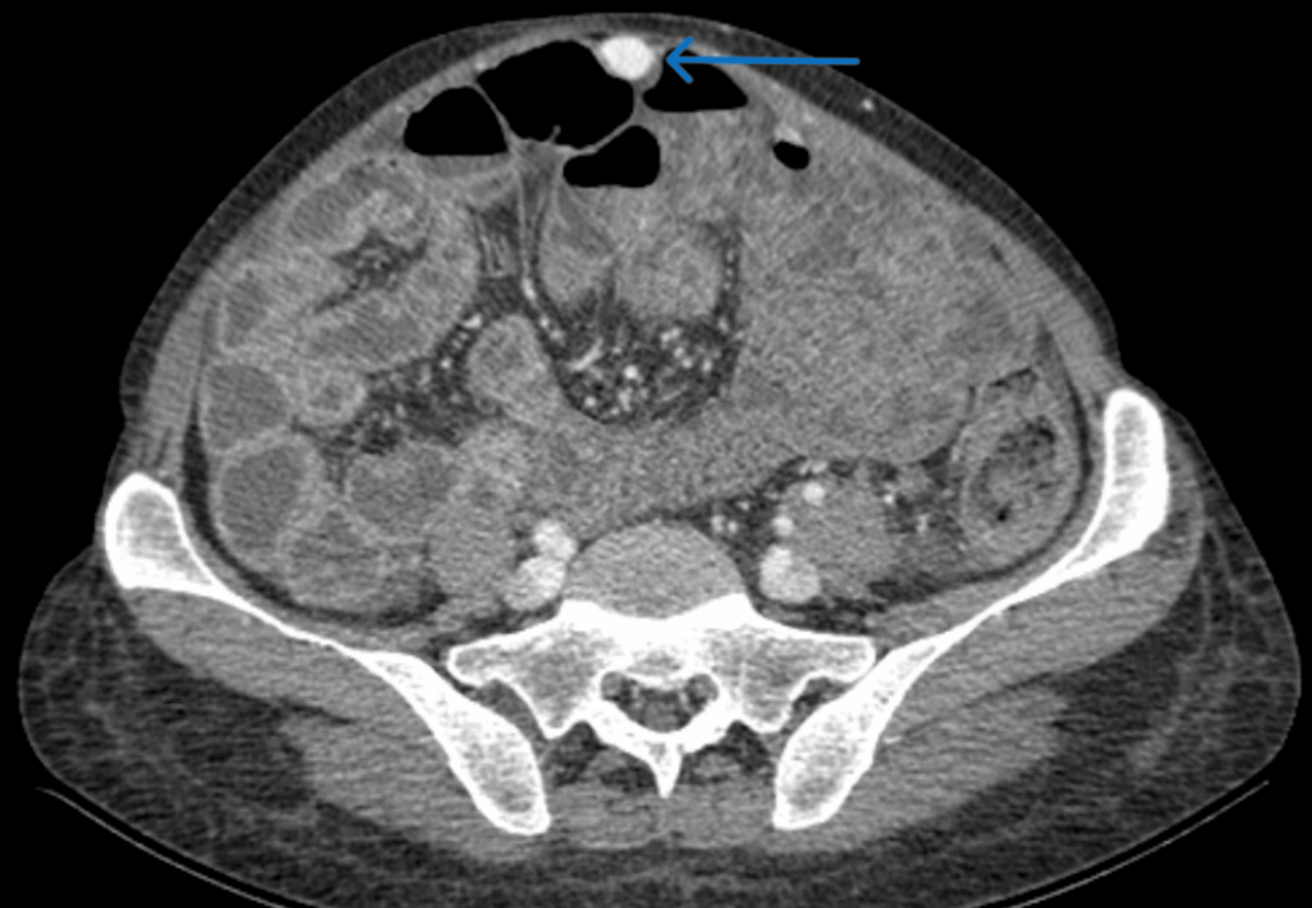
Portal-phase abdominal CT showing the superficial component of the shunt running within the anterior abdominal wall (blue arrow)

**Figure 4:**
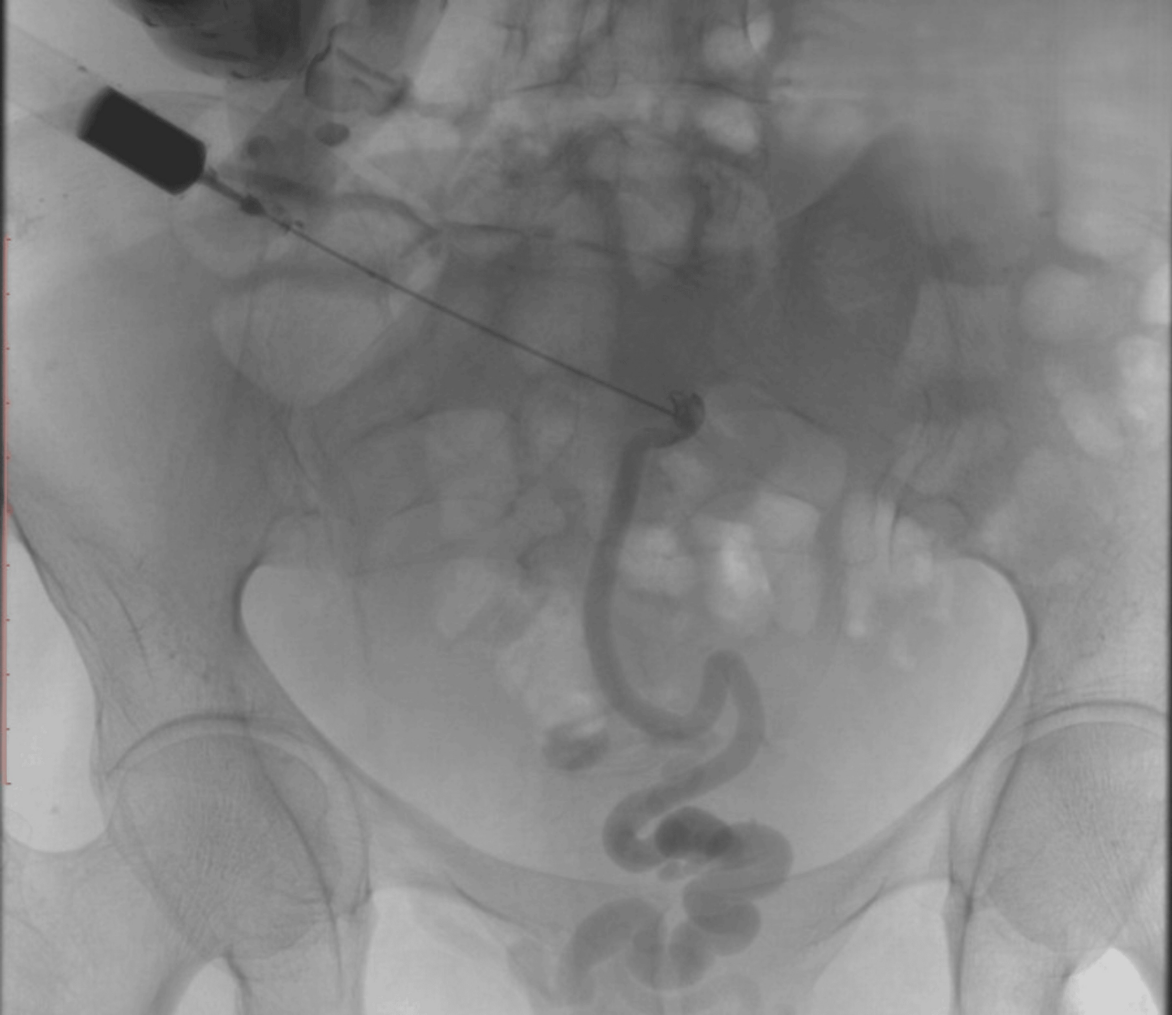
Venogram obtained after percutaneous access of the efferent vein

After blood transfusion and initiation of vasoactive therapy, the superficial component of the shunt running within the anterior abdominal wall (Figure 3) was percutaneously accessed under ultrasound guidance (Figure 4). Retrograde embolization of the varices was then performed using N-butyl-2-cyanoacrylate mixed with ethiodized oil, without the use of balloon occlusion. This procedure achieved immediate bleeding control.

A complete and sustained biochemical response of PBC was achieved following the initiation of ursodeoxycholic acid (UDCA) therapy and adherence to a gluten-free diet. At nine years of follow-up, no hemorrhagic recurrence or additional hepatic events have been observed.

## Discussion

Small bowel varices are a form of ectopic varices that develop in patients with portal hypertension, most commonly at sites of previous abdominal surgery. They arise due to postoperative adhesions that cause focal venous constriction and localized areas of portal hypertension [3,4].

The tributaries of the superior mesenteric vein form the afferent pathway, while the efferent pathway typically involves the anterior abdominal wall veins or the retroperitoneal veins of Retzius, which drain either directly into the inferior vena cava (IVC) or via the iliac, renal, or gonadal veins [5,6].

Bleeding from ectopic varices should be considered in any patient with portal hypertension when the bleeding source remains unidentified after upper endoscopy and colonoscopy. A history of previous abdominal surgery should further raise the level of suspicion [3-10].

Contrast-enhanced cross-sectional imaging allows accurate diagnosis, detailed delineation of shunt anatomy, and appropriate selection of the treatment approach and venous access route [4].

Treatment options for ectopic varices include endoscopy, surgery, and endovascular therapy [3]. Unlike duodenal or rectal varices, jejuno-ileal varices cannot be reached with conventional endoscopy, making endovascular therapy the mainstay of treatment [3,4]. Endovascular options include TIPS placement or transvenous obliteration. TIPS placement is preferred in patients with liver decompensation, although it may be insufficient alone because it does not address focal venous constriction and localized portal hypertension [4]. Transvenous obliteration can be performed either retrogradely or anterogradely. In small bowel varices, the retrograde approach is rarely feasible because a single major outflow vein that is readily accessible through systemic venous routes (e.g., the internal jugular or common femoral vein) is uncommon [4]. In contrast, the anterograde approach often represents the only viable option. It involves catheterizing the afferent vein via percutaneous transhepatic or transsplenic access. When a recanalized paraumbilical vein is present, direct puncture of its superficial abdominal wall segment offers a less invasive alternative [4,10]. Whenever anatomically feasible, superficial venous puncture should be considered the first-line approach for transvenous obliteration, as it provides optimal access with reduced morbidity [4,10]. In patients with a TIPS, the anterograde approach can also be performed through the shunt via internal jugular vein access [4]. Retrograde embolization through direct percutaneous puncture of the efferent abdominal wall vein is an uncommon but minimally invasive technique for small bowel variceal obliteration. This approach was used in our case because of the superficial course of the shunt. Saad et al. previously described and supported a similar percutaneous method for managing parastomal varices, which are also superficial adhesion-related mesenteric varices [11]. In our case, a mixture of N-butyl-2-cyanoacrylate and ethiodized oil was used. N-butyl-2-cyanoacrylate polymerizes immediately upon contact with blood, eliminating the need for balloon or vascular plug occlusion to prevent systemic diffusion [2].

## Conclusions

Small bowel varices are a distinct subtype of ectopic varices that most often develop after prior abdominal surgery. Diagnostic delays are common due to their deep location within the gastrointestinal tract and limited clinician awareness. Ectopic varices should be considered in any patient with portal hypertension who presents with unexplained gastrointestinal bleeding, particularly following abdominal surgery. Contrast-enhanced cross-sectional imaging is essential for diagnosis and treatment planning. Transvenous obliteration remains the mainstay of therapy, though obtaining suitable venous access can be challenging. When anatomically feasible, superficial venous puncture should be the preferred first-line approach for transvenous obliteration because of its minimally invasive nature.
